# A Proposed Bill to Regulate the Practice of Medicine in Michigan

**Published:** 1882-12

**Authors:** Henry B. Baker

**Affiliations:** Lansing, Michigan


A PROPOSED BILL TO REGULATE THE PRACTICE OF MEDICINE IN MICHIGAN. BY HENRY B. BAKER, M. D., LANSING, MICHIGAN. (Reprint from the Michigan Medical News, October 10, 1882.)

I submit herewith a copy of my proposed bill to regulate the practice of medicine, first published in the *Physician and Surgeon*, Ann Arbor, Mich., November, 1880, but here somewhat amended, attention having been given to some suggestions made by persons who have also given the matter careful thought.

Some have proposed that the State accept diplomas, as they are accepted by the State Board of Health of Illinois; but it seems to me that the public can well take advantage of the natural rivalry of the colleges, and thus elevate the standard of medical education by a law which shall practically set each college in Michigan to watch each other one in this State, and all others whose graduates shall practice here. Every college would have a greater inducement than now to have its graduates creditable to it; because some of its graduated would be publicly tested and compared with those of other colleges.

Objection has been made that by this bill the great body of physicians in the State would have no voice in the proposed examining board. To this it may be replied that they have no voice now; and that this bill is not drawn for the especial and exclusive benefit of physicians, but is from the standpoint of one endeavoring to act for the interests of the general public. It is believed that the public would have had protection long ago if it had not been for the unconquerable disagreements among medical practitioners. It is not probable that the several thousands of medical proctitioners in Michigan will soon agree upon a plan; because they include all shades of belief and all degrees of intelligence, from the "medicine man" up to the highest type of physician known. But if some such law as is here proposed shall be enacted and enforced for one generation, the next generation of physicians will probably be of a type fit to control the interests of the public in relation to the medical profession.

But while this bill was not drawn for its direct benefits to physicians, its author believes that, if enacted into law, it is likely to accomplish what all the foremost physicians have professed to seek, namely, the advancement of the standard of those who profess and practice medicine. Let any person who questions this study the nature of the examination proposed in section 9, and consider that it is to be participated in by representatives of colleges from which many of the leading physicians in this State graduated, that results of the examinations will become to a certain extent public property, and ask himself whether a majority of the persons now actually practicing medicine in this State were ever qualified to pass such an examination.

The bill does not propose to interfere with any person now practicing in Michigan; but it proposes that, after the act passes, no unqualified person shal. begin to practice medicine in this State. My view is that by such a regulation, the number of ignorant doctors would soon be greatly lessened, and the dignity and honor of the medical profession greatly promoted; and this implies greatly increased safety of human life.

### THE PROPOSED BILL.

SECTION 1. *The people of the State of Michigan enact*, That, from and after the time when this act shall take effect, no person shall begin the practice of medicine or of any branch or department thereof (except dentistry), or profess to begin the practice thereof, in this State, without first exhibiting evidence of qualifications for such profession in accordance with the provisions of this act. In this section, the term "begin the practice of medicine in this State," shall not apply to any person who at the time of the passage of this act is actually practicing medicine in this State; *provided*, that such person shall have registered as a practicing physician, as provided in this act.

SECTION 2. A State Board of Medical Examiners is hereby constituted as follows: The faculty of each legally-constituted and reputable medical college in this State, authorized by law to confer the degree of Doctor of Medicine, and actually existing and teaching as such a college, shall biennially name one member, the Superintendent of Public Instruction shall biennially name one member, and the State Board of Health shall biennially name one member; of the persons thus named in the first instance, six shall be appointed by the Governor with the advice and consent of the Senate, and when duly qualified, and when their oaths of office shall have been filed in the office of the Secretary of State at Lansing, they shall organize as a State Board of Examiners, and shall elect from their number a president, a secretary, and such other officers as their organization may require, and shall adopt and publish rules for procedure. *Provided*; that the failure of any college, or of all the colleges, to name a candidate for membership shall not cause a failure to organize or continue the Board; but the Governor or the Governor and Senate shall appoint the six members in the first instance, and two members biennially thereafter, and those actually nominated, appointed and legally qualified, shall organize and perform all the duties of the State Board of Medical Examiners; *Provided* further, that no teacher, professor, lecturer, or officer of any of the before-mentioned colleges, shall be eligible to membership except as a representative to the college to which he belongs. Any vacancy in the Board may be filled, until the next regular session of the legislature, by the Governor.

SECTION 3. The term of office of each member first appointed shall be decided by lot, so that the term of two members shall expire every two years; and the term of office of each member appointed thereafter shall be six years.

SECTION 4. It shall be the duty of the State Board of Medical Examiners to organize as a Board immediately after this act takes effect, and proceed at once to prepare plans for a record-book for the use of the county clerks, blank forms for returns by the county clerks to the State Board of Medical Examiners, and such other blanks, circulars, instructions, etc., as may be necessary to carry this act into effect in the first instance, and with a view to its continuance, and to cause such record-books, blank forms, and circulars to be made by the State printers and binders, and to cause to be given to the Board of State Auditors, for audit and payment out of the general fund, bills for such printing and binding, duly certified by the State printers and binders and by the Secretary of the State Board of Medical Examiners.

SECTION 5. The expenses of the State Board of Medical Examiners, and the compensation of its members, shall be paid out of money collected by the Board from the candidates examined, in accordance with section 6 of this act; *Provided*, that the expenses of starting the work of registration of physicians shall be paid as specified in section 4 of this act.

SECTION 6. Each candidate for examination shall pay to the Board or its treasurer, the sum of —— dollars.

SECTION 7. The State Board of Medical Examiners shall keep a record of all examinations made by it, which record shall include statements of items requisite for identification of the person examined; such as the name, age, sex, color, height, color of hair, color of eyes, and other items if necessary; the names of the examiners, specifying the one who examined each candidate in each subject, the subjects in which each candidate passed successfully, those in which he failed to pass, and his standing in each subject.

SECTION 8. In each year the State Board of Medical Examiners shall make a report to the Governor, which report shall include accounts of receipts and expenditures by the Board, statements of the number of candidates examined, the number and names of those passed and the number rejected, copies of the questions asked—which shall not all be the same in any two years—and copies of rules and regulations of the Board of Examiners; also the number of registered practitioners in each county, as reported to the Board by the county clerks.

SECTION 9. It shall be the duty of the State Board of Medical Examiners to examine each candidate for the practice of medicine or of any branch of the medical practice, as to proficiency in the English language and in the sciences of anatomy, phisiology, pathology, ætiology, chemistry and toxicology, as follows: Relative to the English language, the examination of each candidate shall be such as to ascertain whether the candidate has sufficient intelligence and education to enable him to read and write understandingly, accurately and logically, on such topics as are likely to arise in the course of his studies and in his relations with those who will fill his prescriptions, act as nurses, be his patrons, or officially consider his testimony in court. Relative to anatomy the examination shall be such as to ascertain whether the candidate has sufficient knowledge of the subject to enable him or her to explain the nature and relative position of the different structures in any part of the body, with reference to an injury, surgical operation, or other practical question connected with any of the several branches of medical practice. With reference to physiology, the examination shall be such as to ascertain whether the candidate is able to explain the normal function of each important organ in the human body, so far as the same is known and established in science. With reference to pathology, the examination shall be such as to ascertain whether the candidate is able to explain to one familiar to the science the usual changes which occur in the structures and functions of the different organs, systems and parts of the human body in each of the common diseases. The examination in ætiology shall extend to what is known of the causes of the principal diseases which prevail in this State. The examination in chemistry shall include tests of the candidate's knowledge of the characters of acids, bases, alkaloids, alcohols and ethers, the reactions which occur under given circumstances between different chemicals or substances used as medicines, the chemistry of the blood and other fluids and solids of the human body, the proximate analysis of urine, the normal composition of cow's milk, and the chemistry of foods, nutrients and drinks. The examination in toxicology shall be sufficient to indicate the candidate's knowledge of the most common poisons, the nature and appearance of each, the common sources of each, the effects upon the healthy human being, poisonous and fatal doses of each poison, tests for poisons, and the chemical and household antidotes for the poisons. In each of these examinations questions upon which only opinions can be expressed shall not be asked; but in examinations in the English language the questions shall be restricted to established usage, and in the sciences they shall be restricted to established and demonstrable knowledge, accepted as such by those who teach those sciences.

SECTION 10. The State Board of Medical Examiners shall grant to candidates who pass a satisfactory examination in the several subjects required by section 9 of this act, a certificate, under the seal of the Board, stating when they were examined and in what subjects they passed an examination. Provided that the Board may decline to examine or to grant a certificate to any candidate whose moral character is bad.

SECTION 11. It shall be the duty of the clerk of each county in this State to receive the books, instructions, blank forms for returns, etc., prepared in accordance with section 4 of this act; to make and keep a record of all physicians entitled to be recorded under this act; and to report annually to the State Board of Medical Examiners, at Lansing, the names, post-office addresses, etc., of physicians recorded during the year, and such other facts as are required in the instructions of the State Board of Medical Examiners, in order to fulfill the intent of this act.

SECTION 12. Any person who at the time of the passage of this act is actually practicing as a physician in this State, may, at any time within ——— months after this act shall take effect, apply to the clerk of the county in which he resides, to be recorded as a physician, in manner as follows: The application shall be on a blank prepared by the State Board of Medical Examiners, and supplied by the county clerk, and shall specify the time and place of such practice, the "school," pathy, specialty, or system of therapeutics or other branch of medicine practiced, the time and place of graduation as a doctor of medicine, the age, sex, color, place of residence and post-office address of the applicant, and such other facts as the State Board of Medical Examiners shall deem important for purposes of identification or to be placed upon record, and shall provide for in the blank forms or instructions prepared by such State Board.

SECTION 13. Upon the receipt of an application as specified in section 12, duly signed and sworn to by the applicant, and attested by two witnesses before some person known to the county clerk to be authorized to administer such oaths, the county clerk shall make the record contenplated by this act; *provided* such sworn application shall show that the applicant has practiced medicine, as specified in section 12, for the time and as otherwise required by this act, in order to authorize a person to practice medicine in this State; and *provided* further, that the application shall reach the county clerk within ——— months after this act takes effect. A practitioner concerning whom such record is made, shall be considered a legally-registered practitioner of medicine in that county.

SECTION 14. At any time after this act shall take effect, upon receipt of a certificate issued by the State Board of Medical Examiners, stating that the applicant has passed the examination required to be made by the Board, together with an application similar to that specified in sections 12 and 13, including also a similarly sworn and attested statement that the applicant is the same person examined, and to whom the certificate was issued, the county clerk shall make the record of the applicant as a practitioner of medicine, and such practitioner shall be authorized to practice medicine in that county.

SECTION 15. In case a practitioner removes from one county to another, it shall be the duty of the practitioner to place upon record with the clerk in the county from and the county to which he removes, his correct post-office address, and to cause a certified copy of the original record to be made and recorded as specified in this section. A practitioner may be recorded in as many counties as he pleases, but his post-office address and residence must, in each case, be specified. Upon application, together with a copy of the record of himself as a practitioner, certified by the clerk of a county to be an accurate and complete copy of the original record, together with a sworn and attested statement by the applicant that he is the person described in the original record, and that he intends to practice medicine within the county in which he thus applies and other than that in which the record was first made, the clerk of the county shall make and keep a record, which shall include the first record, and such other facts as the State Board of Examiners shall specify in its instructions.

SECTION 16. A certified copy of the record of any practitioner may be obtained by any person from the county clerk upon payment of the proper or the usual fee for such service.

SECTION 17. No person who practices medicine, surgery or midwifery, in any of their branches (except dentistry), shall be able in any of the courts of this State to collect pay for professional services rendered subsequently to the time stated for this abt to go into effect, unless he or she was, at the time such professional services were rendered, duly registered as a medical practitioner according to the several provisions of this act.

SECTION 18. Whoever violates a provision of this act shall forfeit a sum not exceeding ——— dollars. It shall be unlawful for any person to practice, or profess to practice medicine, surgery, obstetrics, or any art of healing, or treatment of the sick (except dentistry) or receive pay for practicing in this State, except there shall be in the office of the clerk of the county in which the practice is performed, a record of such person as a practitioner, in accordance with the several sections of this act; and if any such person who has not complied with this act, and who has not been thus authorized, shall practice or profess to practice medicine, surgery, or the art of healing, or any of their branches, whether of therapeutics, obstetrics, surgery, or any other department thereof (except dentistry), in this State, he or she shall, be deemed guilty of a misdemeanor, and on conviction thereof, shall be liable to a penalty in any sum not exceeding ——— dollars, and to imprisonment not exceeding ——— years.

SECTION 19. It shall be the duty of the State Board of Health, and of the health officer and local board of health in each township, city and village, to co-operate with the State Board of Medical Examiners, which is hereby charged with the duty of securing the fulfillment of the requirements of this act.

SECTION 20. The Board of State Auditors shall, if required, provide and furnish office-room at Lansing for the State Board of Medical Examiners.

